# *Pseudempleurosoma haywardi* sp. nov. (Monogenea: Ancyrocephalidae (*sensu lato*) Bychowsky & Nagibina, 1968): An endoparasite of croakers (Teleostei: Sciaenidae) from Indonesia

**DOI:** 10.1371/journal.pone.0184376

**Published:** 2017-09-07

**Authors:** Stefan Theisen, Harry W. Palm, Sarah H. Al-Jufaili, Sonja Kleinertz

**Affiliations:** 1 Aquaculture and Sea-Ranching, University of Rostock, Rostock, Germany; 2 Centre for Studies in Animal Diseases, Udayana University, Badung Denpasar, Bali, Indonesia; 3 Laboratory of Microbiology Analysis, Fishery Quality Control Center, Ministry of Agriculture and Fisheries Wealth, Al Bustan, Sultanate of Oman; Charles University, CZECH REPUBLIC

## Abstract

An endoparasitic monogenean was identified for the first time from Indonesia. The oesophagus and anterior stomach of the croakers *Nibea soldado* (Lacépède) and *Otolithes ruber* (Bloch & Schneider) (n = 35 each) sampled from the South Java coast in May 2011 and *Johnius amblycephalus* (Bleeker) (n = 2) (all Sciaenidae) from Kedonganan fish market, South Bali coast, in November 2016, were infected with *Pseudempleurosoma haywardi* sp. nov. Prevalences in the first two croakers were 63% and 46%, respectively, and the two *J*. *amblycephalus* harboured three and five individuals. All three croakers represent new hosts for this monogenean genus. We provide infection rates, light microscopical observations, 3D confocal microscopical illustrations, and a morphometric comparison with all congeners. The new species differs in body size, the position and shape of the ovary and testes, and especially in the composition of the dorsal anchor complex, with the dorsal bar being anteriorly concave rather than planar or convex as in its congeners. The dorsal and ventral anchors of this new species are the longest in the genus, whereas the male copulatory organ is the smallest. The first DNA sequences for a member of this genus demonstrate the greatest similarity with endoparasitic freshwater monogeneans from African cichlid fishes. This suggests a freshwater origin for these marine endoparasitic monogeneans.

## Introduction

The Monogenea are common ectoparasitic flatworms of fish, usually infecting gills, fins and scales, but also the eyes and nostrils. They feed on blood, mucus or epithelial cells of their host. The group is highly diverse, with an estimated 25,000, usually highly host specific, species. Besides the unique haptor (posterior attachment apparatus), the monoxenous life cycle is a typical feature which contrast to other platyhelmiths. The adults are hermaphroditic and reproduce sexually, with eggs releasing ciliated oncomiracidiae larvae; these seek a fish host, mainly photo- and chemotactically, and develop into adults. The combination of a direct life cycle, high host specificity and the adverse effects due to feeding on fish inducing possible secondary bacterial or viral infections often leads to mass infections in finfish aquaculture. They have resulted in disastrous financial losses worldwide (e.g. in commercially important grouper, barramundi, amberjack and salmon farms) [1,2].

Very few monogeneans are endoparasitic, i.e. found inside the host’s body. Occasionally species infect the digestive tract, heart musculature or blood vessels [3]. Species belonging to the genus *Pseudempleurosoma* Yamaguti, 1965 exhibit such a specialization in terms of their infection site that they have adapted to an endoparasitic life, showing different adaptation levels [4], especially in the anchor apparatus, to attach to the oesophageal folds [5]. This type of adaptation is known in only a few genera, such as in the `*Diplectanotrema*-group´, parasitizing the foregut–pharynx, oesophagus and stomach–and rectum of marine fishes, e.g. *Diplectanotrema* Johnson and Tiegs, 1922, *Neodiplectanotrema* Gerasev, Gaevskaja & Kovaleva, 1987, *Paradiplectanotrema* Gerasev, Gaevskaja & Kovaleva, 1987, *Pseudodiplectanotrema* Gerasev, Gaevskaja & Kovaleva, 1987 and *Metadiplectanotrema* Gerasev, Gaevskaja & Kovaleva, 1987 [5]. Additionally, this adaptation is present in monogeneans of the genus *Enterogyrus* Paperna, 1963, with species that inhabit the stomach of freshwater fishes [6,7], and in *Montchadskyella* Bychowsky, Korotajeva & Nagibina, 1970 (Montchadskyellidae, also marine, but pseudosegmented in contrast to the `*Diplectanotrema*-group´) [8]. This leads to the assumption that evolution to an endoparasitic lifestyle happened multiple times in monogenean history. Rarely the urinary bladder of fishes can be parasitized, and even freshwater amphibians and turtles are utilized as hosts (by the genera *Polystoma* (Frölich, 1791) and *Kritskyia* Kohn, 1990, respectively). One out of the thousands of species, namely *Oculotrema hippopotami* Stunkard, 1924 infects the eyes of the hippopotamus (the only known monogenean ectoparasite of mammals) [9–11].

The genus *Pseudempleurosoma* Yamaguti, 1965 was first described for the type species, *P*. *carangis* Yamaguti, 1965, from the hosts *Caranx lugubris* Poey, 1861, *Caranx sexfasciatus* Quoy & Gaimard, 1825 (both Carangidae) and *Myripristis berndti* Jordan & Evermann, 1903 (Holocentridae) from Hawaiian waters (Pacific Ocean) [4]. While monogeneans in general are considered highly host specific, *Pseudempleurosoma* is not, infecting different host species, genera and families. After a re-examination of the holotypes and paratypes of the type species of the genus, which differs from *Metadiplectanotrema* by the presence of two rather than one ventral bars associated with each ventral anchor, and by the presence of an accessory piece around the male copulatory organ (MCO). *Metadiplectanotrema* was considered a junior synonym of *Pseudempleurosoma* [6]. So far, four further species (*P*. *caranxi* (Gerasev, Gaevskaja & Kovaleva, 1987) Santos, Mourão & Cardenas, 2001, *P*. *myripristi* (Gerasev, Gaevskaja & Kovaleva, 1987) Santos, Mourão & Cardenas, 2001, *P*. *gibsoni* Santos, Mourão & Cardenas, 2001 and *P*. *guanabarensis* Carvalho & Luque, 2012) have been described from the Atlantic Ocean, with the additional hosts *Caranx ruber* (Bloch, 1793), *Myripristis jacobus* Cuvier, 1829 (Carangidae resp. Holocentridae), *Paralonchurus brasiliensis* (Steindachner, 1875) (Sciaenidae), *Rachycentron canadum* (L., 1766) (Rachycentridae), *Sphoeroides testudineus* (L., 1758) (Tetraodontidae) and *Trichiurus lepturus* L., 1758 (Trichiuridae) between 1987 and 2012 [5–6,12–13]. All recorded fish host species, except the tetraodontid pufferfish, have an economic value and show similarities in their ecology.

No DNA sequence for members if this group is known, but analyses of phylogenetic relations within the Dactylogyridae, including freshwater endoparasitic taxa, have been provided [14]. It has already been mentioned that endoparasitic monogeneans were dispersed from an origin in freshwater fish with their hosts potentially crossing to the marine environment, while the original ectoparasitic fauna was lost during this migration. It is thought that ectoparasites are not able to tolerate the variation in salinity and osmolarity [14]. For the first time, we now compare sequences of a new marine endoparasitic member of the `*Diplectanotrema*-group’, *Pseudempleurosoma haywardi* sp. nov., with a detailed phylogenetic analysis of most related dactylogyrid taxa, including *Enterogyrus*. We support the hypothesis that marine endoparasitism in monogeneans originates from African freshwater endoparasitic species, and we secure the phylogenetic position of these marine endoparasites. The monogenean species described herein represents a new member within the genus *Pseudempleurosoma*, and is the first species of this genus described from the Indian Ocean. A detailed description using 3D confocal microscopy and a morphometrical comparison between the valid species within this genus are provided.

## Materials and methods

### Sample collection and processing

The sciaenid croakers *Nibea soldado* and *Otolithes ruber* (n = 35 each) were sampled from Cilacap fish market, South Java coast, Indonesia (7°43´25.0´´S 109°01´22.7´´E) in May 2011, and an additional small sample of the sciaenid croaker *Johniusamblycephalus* (Bleeker, 1855) (n = 2) was obtained from Kedonganan fish market, South Bali coast, Indonesia (8°45´25.60´´S 115°10´05.94´´E) in November 2016. The fishes were transferred on ice to the Parasitological and Entomological Laboratory, Biological Faculty, Universitas Jenderal Soedirman (UNSOED University), Purwokerto, Java, and to the Marine and Fisheries Faculty Laboratory, Udayana University (UNUD), Kampus Bukit, Jimbaran, Bali, Indonesia, respectively.

Morphometrical data for each fish were taken (total and standard length (TL and SL) to the nearest 0.1 cm, total and gutted weight (TW and GW) to the nearest 0.1 g). The body cavity was opened and studied by naked eye. The internal organs were transferred to Petri dishes containing NaCl solution (0.9%), and studied for parasites under a Zeiss Stemi DV4 binocular microscope. The endoparasitic monogenean parasites were isolated from the oesophagus/proximal stomach, washed in saline solution and roughly identified under a Novel XSZ-107BN microscope. Some worms were compressed and directly transferred to 95% ethanol for confocal laser scanning microscopy, some worms from each of the three host fish species were directly transferred to 99.8% EtOH for DNA analyses. Most ancyrocephalids were fixed using AFA (Alcohol: Formalin: Acetic acid) or 4% neutral buffered formaldehyde, and then transferred and stored in 70% EtOH for further morphological analyses (light microscopy). Type-specimens are deposited in the Berlin Natural History Museum (ZMB, catalogue Entozoa, holotype: E.7602, paratypes E.7603a-h and E.7604a-i for specimens from additional host) and in the Bogor Zoological Museum, Bogor, Java, Indonesia (paratypes MZBTr 230 (from type host) and MZBTr 231 (from additional host)). The ecological parameters for fish infections with *Pseudempleurosoma haywardi* sp. nov. (prevalence, intensity and abundance) were calculated according to standards [15].

### Light microscopy, drawing and morphological investigation

**Fig 1:**
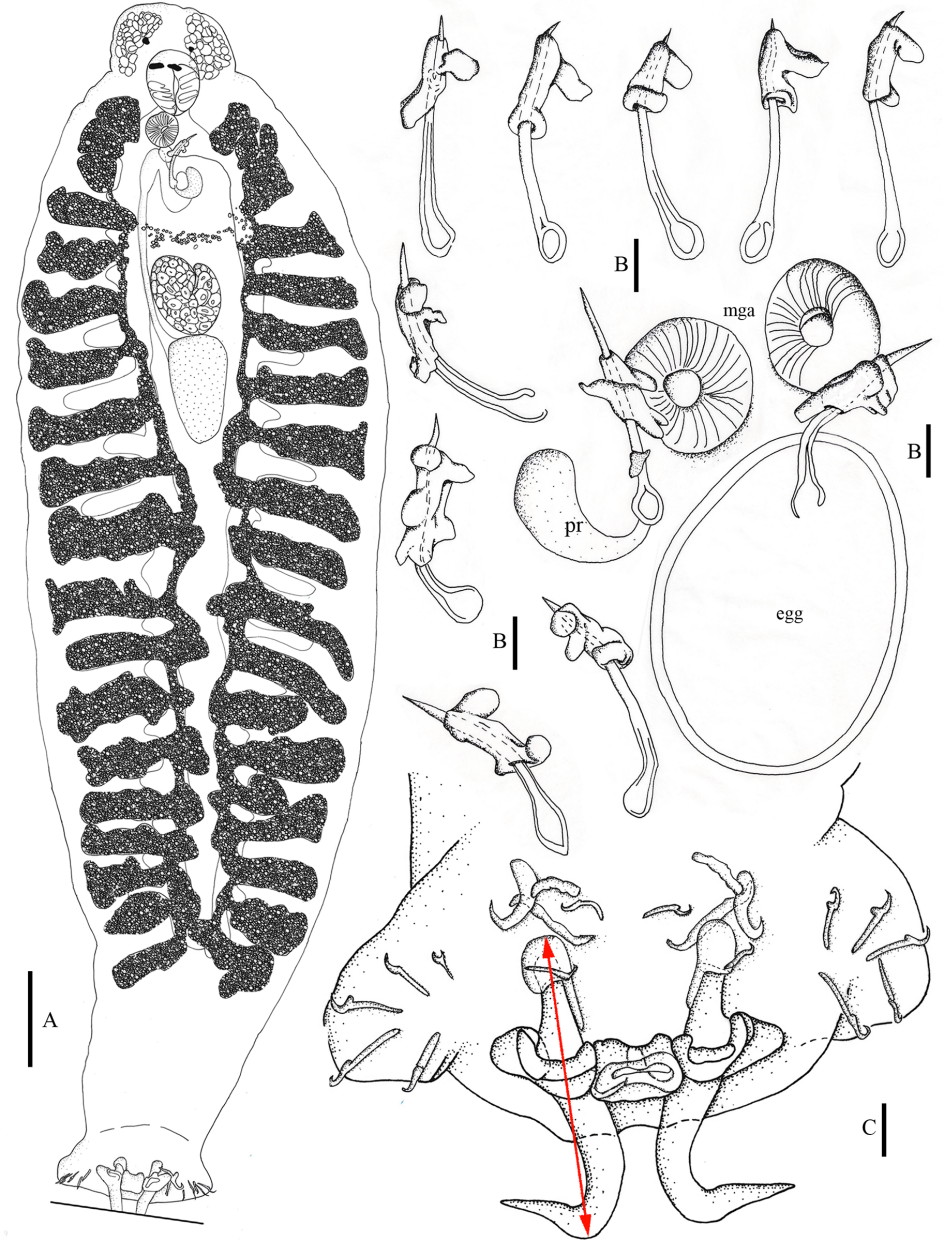
Drawings of *Pseudempleurosoma haywardi* sp. nov. Holotype in ventral view from *Nibea soldado* (**A**), male copulatory organs (MCO) drawings of different *Pseudempleurosoma haywardi* sp. nov. individuals from the fish hosts *Otolithes ruber* (upper row of five MCOs) and *Nibea soldado* (6×), partially shown with position of muscular genital atrium/disc (mga), egg and prostatic reservoir (pr) (**B**) and of the opisthaptor with anchors, bars and seven pairs of hooks (the red arrow defines measuring) (**C**); scale bars **A**: 50 μm; **B** & **C**: 10 μm.

Fixed specimens were cleared and mounted with glycerin following standard parasitological methods [16]. Some whole mounts were also prepared with compressed samples from 70% ethanol and stained with acetic carmine to study the sclerotized hard-parts. Measurements (in micrometres) are according to the initial generic description [4] (red arrow in Fig 1), and are given as the range followed by the mean in parentheses. Illustrations were prepared with the aid of a camera lucida drawing tube. Images were taken with a digital camera (Olympus UC30) attached to an Olympus BX53 light microscope, and measurements recorded with Olympus Cellsens Dimension software, Soft Imaging Solutions GmbH.

### Confocal microscopy

Several 95% ethanol-fixed specimens were processed for confocal laser scanning microscopy following standard procedures [17–18], using a Leica TCS SP2 confocal microscope equipped with an inverted Leica DMIRE2 microscope and a PL APO 363 oil immersion objective (numerical aperture 5 1.4, z section setting minimum 200) at the Live Cell Imaging Center, Department of Biology, University of Rostock.

### DNA analysis

For molecular analysis, genomic DNA was extracted from several adult worms of all three fish host species fixed in 99.8% ethanol following a recent useful protocol [18], i.e. grinding whole worms and using the suspension as a template directly without DNA extraction. Amplification of D1-D3 fragments of the large subunit region (LSU) was achieved using the following primers: C1 (forward; 5’-ACCCGCTGAATTTAAGCAT-3’) and D2 (reverse; 5’-TGGTCCGTGTTTCAAGAC-3’) [14]. The reaction was performed using illustra™ puReTaq Ready-To-Go PCR beads (0.2 ml tubes, 96 reactions), containing 5 pmol of each primer, 5 μl of DNA suspension and nuclease free water to a total volume of 30 μl. Obtained PCR products (1 μl) were viewed on a 0.8% agarose gel stained with ethidium bromide. Contiguous sequences were aligned and assembled using BioEdit v.7.0.9 [19]. The generated sequences (GenBank accession numbers (GBAN) MF115714-MF115717) were aligned with their closest matches in GenBank (23 ingroup and *Actinocleidus recurvatus* (GBAN AJ969951) as an outgroup taxa). Phylogenetic analyses were performed in MEGA version 7 [20] based on the best scoring model (Bayesian Information Criterion, see supporting information S1 Calculation, https://figshare.com/s/75cc37ed9297dc11d983), maximum likelihood was used for the best fitting tree according the General Time Reversible Model, rates among sites were gamma distributed with invariant sites (G + I), the number of discrete gamma categories was 5, and using complete deletion of gaps as gaps missing data treatment. The robustness of the inferred phylogeny was assessed using a bootstrap procedure with 1,000 replications [21].

### Ethic statement

In this study, experiments were not performed on live vertebrates. Instead, freshly caught dead fish was used and therefore no ethics statement is required. Samples were taken within the Indonesian German joint research cooperation “SPICE” (Science for the Protection of Indonesian Marine Coastal Ecosystems), a German Indonesian initiative in earth system research, with research permit from RISTEK, the Indonesian State Ministry of Research and Technology, and in cooperation with Udayana University, Denpasar, Bali. The fishes were obtained from official fish markets (see Material and Methods section), national laws regulate captures and fishermen/salesmen are licensed, sampled fish species are common in Indonesia, not protected, and the number of 35 individuals per species was sampled because it is a good estimate to analyze the entire parasite community of a given fish species at a given location, and 35 is a minimum number for statistical tests.

### Nomenclatural acts

The description of *Pseudempleurosoma haywardi* **sp. nov.** (LSID species identifier number: urn:lsid:zoobank.org:act:828053D6-3445-4BF5-BEEA-71F60411DF6D) complies with the requirements of the International Commission on Zoological Nomenclature (ICZN). The electronic edition of this article conforms to the requirements of the amended International Code of Zoological Nomenclature, and hence the new names contained herein are available under that Code from the electronic edition of this article. This published work and the nomenclatural acts it contains have been registered in ZooBank, the online registration system for the ICZN. The ZooBank LSIDs (Life Science Identifiers) can be resolved and the associated information viewed through any standard web browser by appending the LSID to the prefix “http://zoobank.org/”. The LSID for this publication is: urn:lsid:zoobank.org:pub:330FC71D-07AC-49A8-B5C2-06BE39667B0B. The electronic edition of this work was published in a journal with an ISSN, and has been archived and is available from the following digital repositories: PubMed Central, LOCKSS and ResearchGate. DNA sequences are available in GenBank under the GBAN MF115714-MF115717.

## Results

### Taxonomy and description

Family Ancyrocephalidae (*sensu lato*) Bychowsky & Nagibina, 1968

*Pseudempleurosoma* Yamaguti, 1965

Syn. *Metadiplectanotrema* Gerasev, Gaevskaja & Kovaleva, 1987 ([6])

*Pseudempleurosoma haywardi* **sp. nov.**

urn:lsid:zoobank.org:pub:330FC71D-07AC-49A8-B5C2-06BE39667B0B

*Type-host*: soldier croaker, *Nibea soldado* (Lacépède, 1802) (Sciaenidae)

*Additional hosts*: tigertooth croaker, *Otolithes ruber* (Bloch & Schneider, 1801) (from type-locality) and bearded croaker, *Johnius amblycephalus* (Bleeker, 1855) (from additional locality) (both Sciaenidae)

*Type-locality*: Cilacap, South Java coast, Indonesia (7°43´25.0´´S 109°01´22.7´´E)

*Additional locality*: Kedonganan, South Bali coast, Indonesia (8°45´25.60´´S 115°10´05.94´´E)

*Habitat*: oesophagus/proximal stomach

*Type-material*: holotype E.7602; paratypes E7603a-h (and additional specimens from additional host E7604a-I), and additional paratypes MZBTr 230 and MZBTr 231 (latter from additional host).

*Deposition of specimens*: Berlin Natural History Museum, Germany (“Museum für Naturkunde”) (holotype E.7602; paratypes E7603a-h and additional specimens from additional host E7604a-I), and Bogor Zoological Museum (“Museum Zoologicum Bogoriense”), Indonesia (paratypes MZBTr 230 and specimens from additional host MZBTr 231).

*Infection*: 22 of 35 fish (*N*. *soldado*) harboured 65 specimens and 16 of 35 fish (*O*. *ruber*) examined harboured 32 specimens. Prevalence 63% (*N*. *soldado*) and 46% (*O*. *ruber*), mean intensity 3 (*N*. *soldado*) and 2 (*O*. *ruber*), intensity 1–7 (*N*. *soldado*) and 1–6 (*O*. *ruber*), mean abundance 2 (*N*. *soldado*) and 1 (*O*. *ruber*). The two *J*. *amblycephalus* from Bali were infected with three and five individuals respectively.

*Etymology*: the specific name is for Dr. Craig J. Hayward who recorded the first unidentified *Pseudempleurosoma* nearby Indonesian waters in 1997.

**Fig 2:**
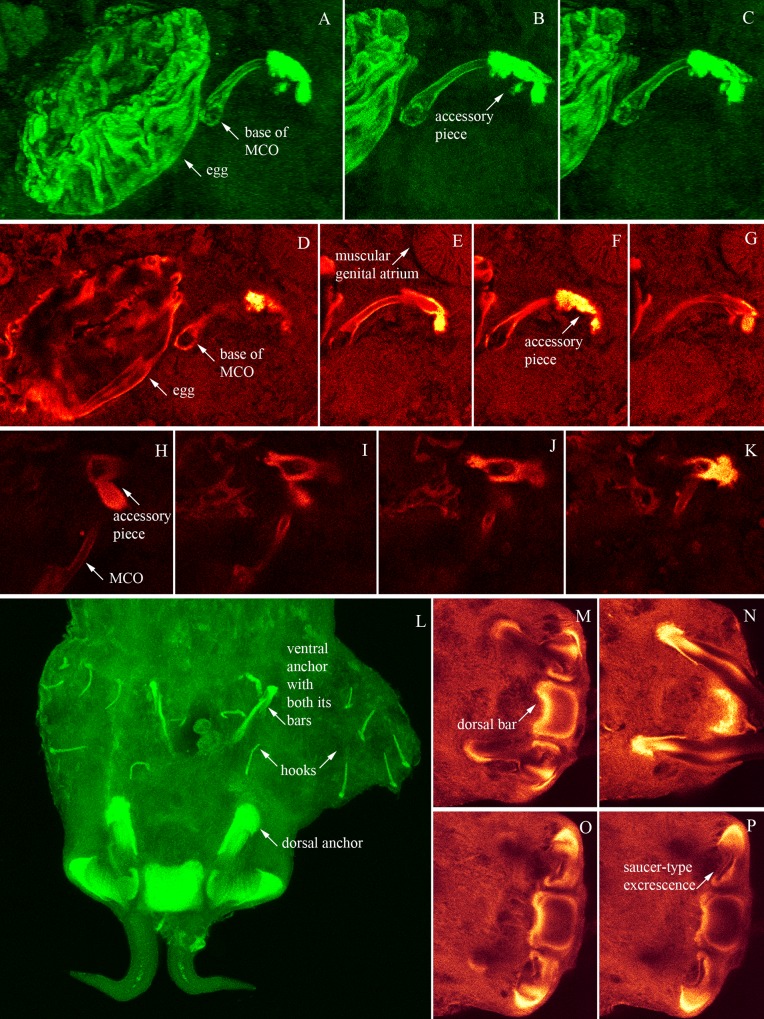
Confocal photos of *Pseudempleurosoma haywardi* sp. nov. Confocal microscopy illustrations of male copulatory organ (MCO) of *Pseudempleurosoma haywardi* sp. nov. with accessory pieces (**A-K**), from different angles (**A-C**), at different levels (**D-G**) and from a second worm (**H-K**), and of the opisthaptor with its hooks, anchors and bars (dorsal bar concave anterior) (**L**) as well as from different levels of the dorsal anchor apparatus (outer root extension with chitin containing cap, inner root extension with saucer-type excrescence) (**M-P**); additional confocal photos are shown in the supporting information S1 Fig, https://figshare.com/s/75cc37ed9297dc11d983.

*Description* (all measurements in μm) (Figs 1 and 2): based on 12 specimens from the type host *N*. *soldado* (see also supporting information S1 and S2 Tables and S1 Fig for details, https://figshare.com/s/75cc37ed9297dc11d983).

Body slender, unspined, 588–1295 (971) long; maximum width 181–361 (289) (at level of boarder ovary/testis). Single pair of head glands extends to pharyngeal region. Two pairs of eye-spots. Oral aperture ventral; pharynx globular, 40–67 × 40–63 (52 × 48) (length × width) (Fig 1A). Intestinal caeca overlaid posterior to testis, coexisting with vitelline follicles, with lateral diverticula lacking haematin pigment. Haptor, 53–84 × 101–142 (66 × 116) length × width), not distinctly set off from body, but laterally lobed; with 2 pairs of dissimilar anchors and 14 marginal hooks (Figs 1A, 1C and 2L). Dorsal anchors, measured according to genus original description [4] from tip of ventral root to height of curve of blade (with inner dorsal root being shorter than outer ventral root in this genus), comparatively long (longest in genus), 59–61 (60) in length, connected by single quadrangular dorsal bar with distinct concave anterior border, 12–21 (19) from end to end, with distance of 12–17 (15) from anterior to posterior border (Figs 1C and 2L–2P). Ventral anchors 14–16 (15) in length, each one with two bars: one attached of 8–18 (11) in length and one free irregular bar of 17–21 (19) in length (Figs 1C and 2L). Marginal hooks 13–16 (15) long (Figs 1C and 2L). Male copulatory organ (MCO) sclerotized, tubular, 29–51 (42) long, with seminal vesicle and prostatic reservoir at base; accessory piece sclerotized, irregular in shape, 14–23 (20) long (Figs 1B and 2A–2K). Testis post-ovarian, oval to almost triangular, widest at its anterior region, 39–95 × 26–57 (76 × 41) (length × width) (Fig 1A). Ovary turned back on itself, giving appearance of being oval, 44–101 × 32–74 (77 × 55) (length × width) (Fig 1A). Uterus sacciform, ends as muscular aperture of 21–39 × 20–31 (29 × 25) (length × width), in smooth genital atrium, almost round in shape (Fig 1A and 1B). Vagina simple, opens at level of muscular genital atrium, no sclerotization visible even in laser confocal microscopy. Vitelline follicles arranged longitudinally in lateral fields along body (Fig 1A). Vitelline ducts unite in a slender line half way between the base of MCO and ovary. Eggs oviform, 56–72 × 39–59 (67 × 50) (length × width), polar filament absent (Figs 1B and 2A).

### Remarks

Due to the presence of i) an accessory piece on the MCO and ii) two bars associated with each ventral anchor, as well as iii) one bar connecting the two dorsal anchors, the new species belongs to *Pseudempleurosoma* (see Table 1 and [6]). The specimens from all sampled fish species belong to the same species (Table 2 and in more detail in the additional supporting information S1 Table and S2 Table), and thus measurements of *P*. *haywardi* sp. nov. from both host species sampled in higher number (n = 35, see Material and Methods section,) are combined for the following comparison with its congeners (based on 12 specimens from the type host *N*. *soldado* and 11 specimens from the additional host *O*. *ruber*).

**Table 1:** Key characteristics of the genera within the “*Diplectanotrema*-group.”

Genus	Accessory piece on MCO	Ventral anchors with bars
*Diplectanotrema*	Yes	No
*Neodiplectanotrema*	No	Yes, with one shared
*Paradiplectanotrema*	Yes	Yes, with one each
*Pseudodiplectanotrema*	No	No
Pseudempleurosoma*	Yes	Yes, with two each**

*Synonym: *Metadiplectanotrema* [6]

**One bar was stated in the initial generic description [4], but re-examination [6] showed that there are two bars, and "anterior pair of hooklets [= hooks (authors)] large" [4] probably means the second ventral bars. Another interpretation stated no bar within the documentation of a single South East Asian individual [22] (see Discussion); MCO: male copulatory organ

**Table 2:** Comparative linear measures for *Pseudempleurosoma* spp.

*SPECIES/REFERENCE*	*P*. *carangis* [4]	*P*. *caranxi* [5,6]	*P*. *myripristi* [5,6]	*P*. *gibsoni* [6]	*P*. *guanabarensis* [12]	*P*. *haywardi* sp. nov., present study, from *Nibea soldado* (type-host)	*P*. *haywardi* sp. nov., present study, from *Otolithes ruber* (additional host)
*MEASUREMENTS (all µm)*							
**Body (length x width)**	800–1,320 x 150–340	1,440–1,630 x 310–440 (1,550 x 410)	590–1,250 x 220–860 (1,080 x 410)	950–1,540 x 161–308 (1,228 x 222)	1,350–2,750 x 425–850 (2,098 x 690)	588–1,295 x 181–361 (971 x 289)	582–937 x 161–305 (757 x 230)
**Opisthaptor (length x width)**	70–90 (width only)				68–138 x 70–185 (93 x 125)	53–84 x 101–142 (66 x 116)	58–88 x 87–137 (71 x 119)
**Pharynx (length x width)**					65–160 x 35–160 (120 x 119)	40–67 x 40–63 (52 x 48)	44–64 x 42–55 (53 x 47)
**Ovary (length x width)**				46–92 x 46–82 (68 x 70)	80–170 x 75–200 (140 x 167)	44–101 x 32–74 (77 x 55)	40–64 x 28–62 (52 x 39)
**Testis (length x width)**				36–65 x 27–51 (47 x 33)	90–235 x 60–165 (142 x 119)	39–95 x 26–57 (76 x 41)	45–68 x 29–47 (56 x 34)
**Dorsal anchor**	47–53	43?-broken	46–55 (48)	41–58 (46)	38–58 (52)	59–61 (60)	58–64 (61)
**Dorsal bar (length x width)**	12.5–15 (length only)	17 x 17.5	21.5 x 18	12–16 x 14–18 (14 x 16)	23–28 x 10–18 (25 x 13)	12–21 x 12–17 (19 x 15)	19–20 x 12–17 (20 x 15)
**Ventral anchor**	10–15	12.5	11–12.5 (12)	12–14 (12)	10–14 (12)	14–16 (15)	14–18 (16)
**Attached ventral bar**	15	17	11–15 (14)	12–16 (14)	5–10 (7)	8–18 (11)	10–16 (13)
**Detached ventral bar**		17.5	12–22 (18)	14–23 (17)	8–13 (11)	17–21 (19)	13–20 (17)
**Marginal hooks (14 pcs)**	10–15	12.5–15 (14)	12.5	14–18 (15)	8–15 (12)	13–16 (15)	13–16 (15)
**Male copulatory organ (MCO)**	50 twisted	49–55 (52) x 1.5	53–58 (56) x 2.0	43–62 (51)	45–70 (59)	29–51 (42)	33–52 (45)
**Accessory piece of MCO**					18–40 (29)	14–23 (20)	15–19 (17)
**Muscular genital atrium (length x width)**	21–27 x 23–25	Present	Present	18–30 x 23–27 (24 x 24)	170–290 (207) (from anterior end)	21–39 x 20–31 (29 x 25)	20–26 x 17–24 (22 x 20)
**Egg (length x width)**	90 x 70	66 x 55	87.5 x 73	78–110 x 58–97 (92 x 78)	75–100 (90) x 40–75	56–72 x 39–59 (67 x 50)	49–78 x 33–59 (68 x 50)
**Filament**	Absent	Absent	37	7–9 (8)	Absent	Absent	Absent
**Hosts**	*Caranx lugubris*, *C*. *sexfasciatus*, *Myripristis berndti* (& *Sphoeroides testudineus* by [13]) (Carangidae, Holocentridae (& Tetraodontidae))	*Caranx ruber* (Carangidae)	*Myripristis jacobus* (Holocentridae)	*Paralonchurus brasiliensis*, *Rachycentron canadum* (Sciaenidae, Rachycentridae)	*Trichiurus lepturus* (Trichiuridae)	*Nibea soldado* (Sciaenidae)	Otolithes ruber* (Sciaenidae)
**Geographical area**	Pacific: Off Hawaii (& Atlantic: East Mexico)	Atlantic: Off Cuba	Atlantic: Off Cuba	Atlantic: Off Brazil and East Mexico	Atlantic: Off Brazil	Pacific: Off South Central Java, Indonesia	Pacific: Off South Central Java, Indonesia

The body length of *Pseudempleurosoma haywardi* sp. nov. is in the range of the body length of *P*. *carangis* and *P*. *myripristi*; *P*. *gibsoni* is slightly longer; *P*. *caranxi* is much longer; and *P*. *guanabarensis* has almost the double body length (see Table 2). The body width of *P*. *haywardi* sp. nov. is similar to the body width of *P*. *carangis* and *P*. *gibsoni*; the body of *P*. *myripristi* is–even though the length is similar to the new species–much wider. The two longer species *P*. *caranxi* and *P*. *guanabarensis* have wider bodies (Table 2). The detached bars of the ventral anchors have similar sizes compared to the congeners’, except *P*. *guanabarensis*, which has smaller detached bars than all other congeners (Table 2). The size of the marginal hooks is similar in all species of this genus (Table 2). Compared to *P*. *haywardi* sp. nov., the sizes of the eggs are larger in all congeners except in *P*. *caranxi*, which shows similar egg sizes (Table 2).

Besides these differences, *P*. *haywardi* sp. nov. differs from all congeners by having longer dorsal anchors. With a length of 58–64 (60), they are the by far longest in the genus. The other five valid species have dorsal anchor length range of 38–58, and mean values of 46–52. Thus, the dorsal anchor length alone is a strong characteristic to distinguish *P*. *haywardi* sp. nov. from its congeners. A second unique characteristic of the new species is the shape of the dorsal bar being anteriorly concave, while all so far described dorsal bars of the congeners are either planar [5] or convex [4,6,12]. Thus, the two structures of the dorsal anchor complex, namely the dorsal anchors and the dorsal bar of *P*. *haywardi* sp. nov., are unique. Also the ventral anchor is the longest in this new species. With sizes of 14–18 (16), it differs from its congeners with only 10–15 (mean around 12). The range of the length of the attached ventral bar of the congeners is similar to the sizes of our material, however, with up to 18, the attached ventral bars of *P*. *haywardi* sp. nov. are also the largest within the genus. In contrast, the male copulatory organ (MCO) is the smallest in the new species, with a mean value of 44, while the other species have mean values of MCO length of 50–59.

Confocal microscopy made it possible to position structures for measuring, providing a range of viewing angles for exact images and measurements, for example a dorsal view on the dorsal bar, as well as focusing through the layers of the dorsal bar from a dorsal view, showing its length and shape accurately.

Other differences from the congeners are the host species, even though a sciaenid host of *Pseudempleurosoma* was already published from the Atlantic (and for a misidentified taxa from Vietnam, probably representing *Pseudempleurosoma*, see Discussion), and the geographic range in Indonesia, South-East Asia.

### DNA analysis

**Fig 3:**
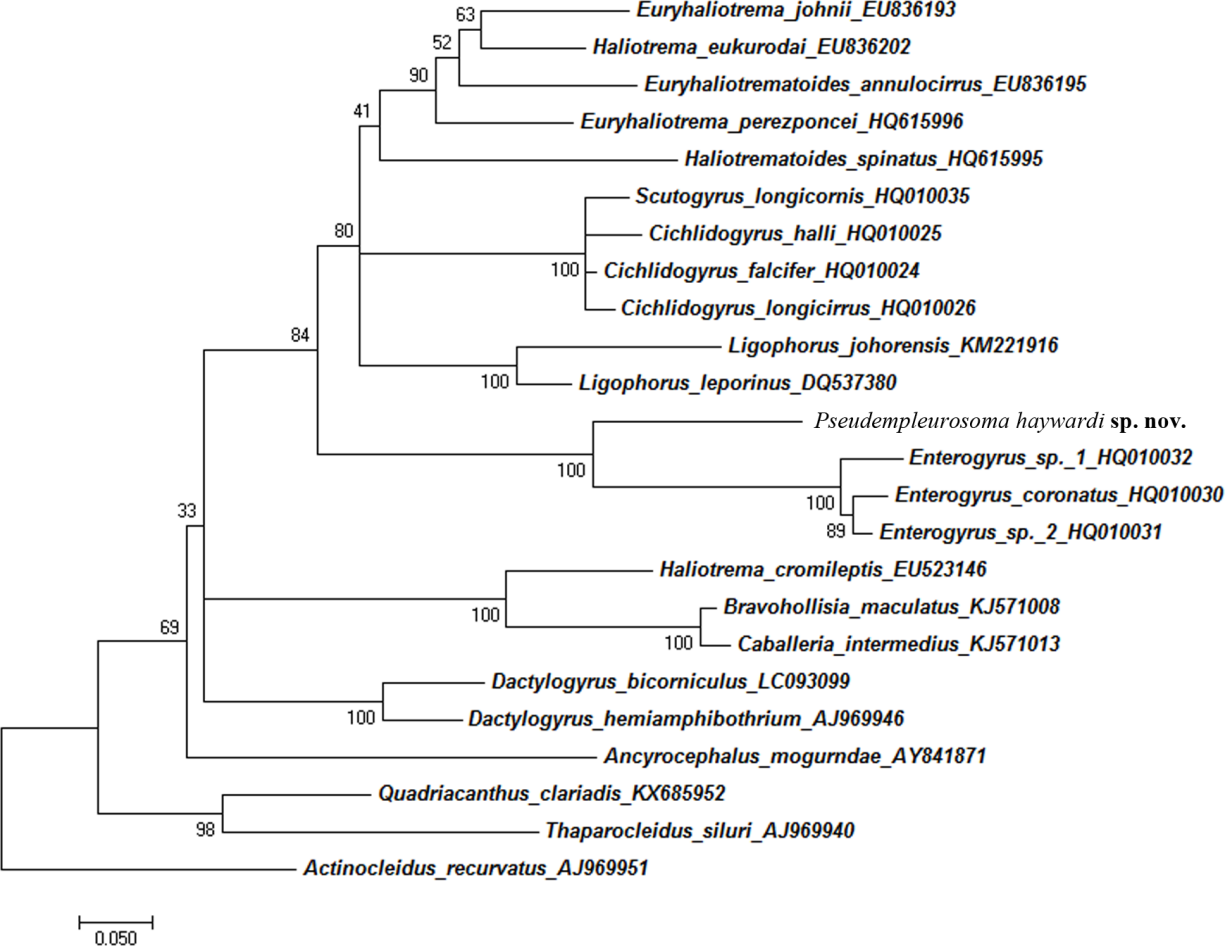
Maximum likelihood tree inferred from the analysis of LSU rDNA. The generated sequences were aligned with their closest matches in GenBank (23 ingroup and *Actinocleidus recurvatus* as an outgroup taxa). Phylogenetic analysis based on General Time Reversible Model with complete deletion used as gaps missing data treatment. The robustness was assessed using a bootstrap procedure with 1,000 replications [20–21]. For sequence details, see S1 Calculation (calculation of best scoring model for phylogeny studies) and S2 Fig (alignment of sequences), https://figshare.com/s/75cc37ed9297dc11d983.

Following a recent protocol [18], we could not amplify the DNA of the worms sampled in 2011, however, analysis of three adult worms collected in 2016 from the bearded croaker *Johniusamblycephalus* led to sequences. The partial LSU rDNA sequences were 840bp long (with primers). A NCBI Genbank blast showed closest similarity to freshwater endoparasitic monogeneans, namely *Enterogyrus coronatus*, *Enterogyrus* "sp. 1 AS-2010" and *Enterogyrus* sp. "2 AS-2010" (GBAN: HQ010032, HQ010031, HQ010030). The new sequence is available under the GBAN MF115714-MF115717. The obtained sequence of *Pseudempleurosoma haywardi* sp. nov. formed a well supported clade with the freshwater endoparasitic Monogenea from cichlid teleosts with a strong bootstrap value of 93% (Fig 3).

## Discussion

### Zoogeography and host range

**Fig 4:**
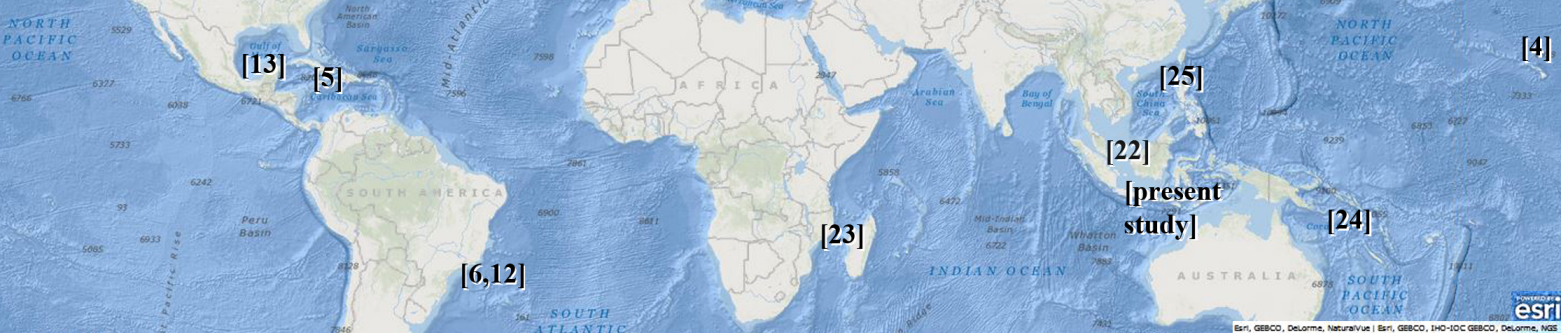
Biogeography of *Pseudempleurosoma* spp. All records of the genus are shown together with the reference, based on English, Russian, Vietnamese and German literature. The record from the Coral Sea [24] is doubtful (see Discussion below) and the record from Vietnam [25] is considered as *Pseudempleurosoma* (see Discussion). Reprinted with permission from Esri Inc. (Environmental System Research Institute) under a CC BY license, original copyright 2017 (see supporting information S1 Permission to use Fig 4), https://figshare.com/s/75cc37ed9297dc11d983.

A summary of the zoogeographical distribution of *Pseudempleurosoma*, ranging from the Gulf of Mexico (*P*. *carangis* [13]) and the Caribbean Sea (*P*. *caranxi* and *P*. *myripristi* [5]) to the South East coast of Brazil (*P*. *gibsoni* and *P*. *guanabarensis* [6,12]), waters off Mozambique [23], the eastern Malaysian Peninsula (unidentified *Pseudempleurosoma* spp. [22,23]), the South Java coast, Indonesia (*P*. *haywardi* sp. nov.), Coral Sea [24] and Vietnam (misidentified *Pseudempleurosoma* sp. [25]) to Hawaii (*P*. *carangis* [4]) is given in Fig 4. The record from the Chesterfield Islands, Coral Sea (unidentified *Pseudempleurosoma* sp. from a hoplichthyid fish [24]), is doubtful (see Fig 4 and Discussion below).

Monogeneans are known being host specific, site selecting and well adapted to their hosts [2,6], but so far members of *Pseudempleurosoma* seem to have a wide host range. The genus has been reported from the Carangidae, Chlorophthalmidae, Holocentridae, Hoplichthyidae, Rachycentridae, Sciaenidae, Sillaginidae and Tetraodontidae [4–6,12–13,23–24] (Tables 2 and 3, Fig 4). Besides the single specimen of *Pseudempleurosoma* sp. from the sillaginid bay whiting *Sillago ingenuua* McKay, 1985 in Malaysia [22], and misidentified worms from the sciaenid croakers *Argyrosomus japonicus* (Temminck & Schlegel, 1843) and *Johnius carouna* (Cuvier, 1830) in Vietnam [25], this is the third record of an endoparasitic marine monogenean from South East Asia. So far the only other record of *Pseudempleurosoma* from a sciaenid, the banded croaker *P*. *brasiliensis*, is from the Atlantic, i.e. the southeastern coast of Brazil [6] (see Tables 2 and 3 and Fig 4). In 2011, an endoparasitic Monogenea was isolated from the stomach of sciaenid croakers *A*. *japonicus* and *J*. *carouna* in Vietnam [25], close to our area of investigation. The geographic area and the closely related host fish species (all Sciaenidae) as well as the provided drawings of the dorsal bar and the anchors and hooks suggest that the worms identified as *Paradiplectanotrema trachuri* belong to *Pseudempleurosoma* with some similarities to *P*. *haywardi* sp. nov.. We herewith establish three new host records for *Pseudempleurosoma*.

**Table 3:** *Pseudempleurosoma* spp. fish host species ecology and economic value. Note that almost all host species are aggregating or schooling, reef-associated or associated to muddy bottoms, and of commercial importance (fish ecology and economy data from [26]).

			*Pseudempleurosoma* species*					
Fish host species	Host ecology	Host economic value	*1*	*2*	*3*	*4*	*5*	*6*
*Caranx lugubris*	Oceanic & insular, also outer **reef** edges, nocturnal, occasionally **schooling**	**Commercial aquaculture**	x					
(Carangidae)								
*Caranx ruber*	Coral **reef** associated, insular & mainland, juveniles in *Sargassum* spp. weed, **schooling**	**Commercial fisheries**		x				
(Carangidae)								
*Caranx sexfasciatus*	Coral **reef** associated, coastal & oceanic, pelagic, nocturnal, stationary **schooling** at daytime, juveniles in estuaries	**Commercial fisheries**	x					
(Carangidae)		** **						
*Myripristis berndti*	Caves & subtidal **reef** flats to outer slopes, benthopelagic, nocturnal, in loose **aggregations**	**Commercial fisheries**	x					
(Holocentridae)								
*Myripristis jacobus*	Shallow coral **reefs** to offshore deep water, nocturnal, **aggregating** on reefs, occasionally swims upside down (parasite?)	Minor			x			
(Holocentridae)								
*Rachycentron canadum*	Mud, sand & gravel bottoms, coral **reefs**, mangroves, estuaries, forms **small groups**	**Commercial aquaculture**				x		
(Rachycentridae)								
*Johnius amblycephalus*	Shallow **coastal waters** and estuaries, rivers (authors: coastal waters in area of distribution are coral reefs, sand and mud bottoms)	Minor						x
(Sciaenidae)								
*Nibea soldado*	Shallow **coastal waters** and estuaries, rivers (authors: coastal waters in area of distribution are coral reefs, sand and mud bottoms)	**Commercial fisheries**						x
(Sciaenidae)								
*Otolithes ruber*	**Coastal waters** (authors: coastal waters in area of distribution are coral reefs, sand and mud bottoms)	**Commercial fisheries**						x
(Sciaenidae)								
*Paralonchurus brasiliensis*	muddy bottoms, near estuaries	Minor				x		
(Sciaenidae)								
*Sphoeroides testudineus*	bays, creeks, seagrass beds, brackish water, rare/absent on coral reefs, forms huge **aggregates**	None	x					
(Tetraodontidae)								
*Trichiurus lepturus*	over muddy bottoms of shallow coastal waters, enters estuaries, **schooling** at bottom, **aggregating** at surface	**Highly commercial**					x	
(Trichiuridae)		**fisheries **						

*1 = *P*. *carangis*, 2 = *P*. *caranxi*, 3 = *P*. *myripristi*, 4 = *P*. *gibsoni*, 5 = *P*. *guanabarensis*, 6 = *P*. *haywardi* sp. nov. (please note that the two additional sciaenid fish species *Argyrosomus japonicus* (commercial fisheries, aquaculture) and *Johnius carouna* (minor commercial) are probably also hosts of *Pseudempleurosoma* [25], see Discussion above)

### Morphological characterization

We compare *Diplectanotrema*, *Neodiplectanotrema*, *Paradiplectanotrema*, *Pseudodiplectanotrema* and *Pseudempleurosoma* (Syn.: *Metadiplectanotrema*) in Table 1. The record from the Pacific (Coral Sea, see above) [24] was described as follows: “a sclerotized copulatory organ without an accessory piece, ventral and dorsal anchor/bar complexes, …and slender shaft”. It was also stated that the genera of the “*Diplectanotrema*-group” are probably synonyms [24]. However, Yamaguti's type specimens of *P*. *carangis* were re-examined and accessory pieces were found [6]. Consequently, these specimens [24] most likely belong to *Neodiplectanotrema* and not *Pseudempleurosoma*, which is considered to have an accessory piece and a ventral anchor/bar complex. The only other genus in the group lacking an accessory piece is *Pseudodiplectanotrema*, but this taxon lacks a ventral bar. In addition, the description of “a dorsal bar formed as two bilateral sclerotized rudiments” [24] does not fit to the so-far described genera of the “*Diplectanotrema*-group”.

The record of *Pseudempleurosoma* sp. [23] from Mozambique did not provide morphometric information, leaving species identification insecure. Measurements were provided for a single specimen from Malaysia [22], revealing similarities to *P*. *carangis*, based on its general morphology, internal anatomy and hooks [22]. However, the morphology of the haptoral complex was misinterpreted [6,22]. According to our confocal images and illustrations, *Pseudempleurosoma* has two dorsal anchors connected by a single bar, combined with two ventral anchors associated with two bars each, one pair connected and one pair disconnected. The original genus description lacked one pair of ventral bars [4], though, re-examination of the type specimens already documented its presence [6], and “three dorsal bars between the large dorsal anchors” (instead of one) combined with no ventral bar were misinterpreted for the Malaysian specimen [22]. However, figure 12 in that publication [22] shows that two of those dorsal bars are saucer-type excrescence root extensions of the dorsal anchors, that were described in more detail for the Ancyrocephalinae [5] as follows: “outer root extension with chitin containing cap, inner root extension with saucer-type excrescence” (in Russian). Comparing the anchor apparatus of *P*. *haywardi* sp. nov. (present study, see Figs 1C & 2L–2P) with the drawings of the generic description with *P*. *carangis* [4], characterizing this saucer-type excrescence as “dorsal root …turned medially to form a dorsal swelling”, and with the drawings of the unidentified specimen [22], it is evident that there is just one dorsal bar in the latter worm as well, as known for *Pseudempleurosoma* spp.. Consequently, this specimen can be affiliated with *Pseudempleurosoma*. This structure was also realized for further species [6], stating: “In *P*. *carangis* and *P*. *gibsoni* …the roots of each dorsal hamulus [= anchor (authors)] are concave, giving the appearance of bars, but in reality there is only one dorsal bar between hamulus.” These saucer-type excrescences, used for distinguishing between species of the Ancyrocephalinae [5] were thus misinterpreted as roots [6] or bars [22]. The description of the Malaysian specimen [22] demonstrates closer similarity to our newly described species compared with *P*. *carangis*. It had an equal length of the dorsal anchors (61 μm), which are largest in *P*. *haywardi* sp. nov. compared to its congeners (mean around 50 μm), and a very long ventral anchor (24 μm). However, this large size results from misinterpretation of the attached ventral bars as belonging to the ventral anchors, while only two (= one pair) of ventral bars, the attached ventral bar of each ventral anchor, were mentioned and drawn in the generic description [4]. The detached, free irregular bars were misinterpreted as “anterior medial hooklets [= hooks (authors)] abnormally large”. In summary, according to the illustration and measurements provided [22] and the explanation given above, this single worm should be affiliated with *Pseudempleurosoma*, is similar to our new species, and their conspecificity so far cannot be excluded.

Other material, probably *Pseudempleurosoma* sp., misidentified as *Paradiplectanotrema trachuri* [25] (Vietnam, see above), differs from the herein described new species. The body length of this *Pseudempleurosoma* sp. (1.7–2.6 mm with a body width up to 824 μm) and the pharynx size are much bigger than of *P*. *haywardi* sp. nov. and in the range of the largest congener, *P*. *guanabarensis*. On the other hand, the MCO of the worms (max. 48 μm) is smaller than in *P*. *haywardi* sp. nov. (max. 52 μm), which was so far considered to have the smallest MCO. The sizes of the dorsal bar, in shape similar to the one of *P*. *haywardi* sp. nov., are in the range of the largest congener, displaying the largest dorsal bar, namely *P*. *guanabarensis*. Dorsal anchors are smaller in this material compared with *P*. *haywardi* sp. nov. and in the congeners range. Detached ventral bars of the Vietnamese specimens are in the same range as in the congeners, except of the largest species, *P*. *guanabarensis*, which displays the smallest detached ventral bars. Attached ventral bars were misinterpreted (thus the worms were misidentified as *Paradiplectanotrema*), and thus measurements are not presented, consequently measurements for the ventral (attached) anchor were misinterpreted as well (however, ventral anchors and attached bars as well as detached bars are visible in the drawing of this worm [25]). The worm still has some similarities with *P*. *haywardi* sp. nov., but differs in several morphological measurements. Again, conspecificity of *P*. *haywardi* sp. nov. and the Vietnamese worm so far cannot be excluded.

Confocal microscopy made it possible to position structures, providing angle of views for illustrating and comparison as well as accurate measurements, for example the saucer-type excrescences, the exact length of the curved MCO, the irregular shape of its accessory piece, the connection of the hooks and bars or the dorsal bar, showing its length, width and shape accurately (Fig 2). This methodology is found to be suitable to detect minor, often difficult to describe differences in the haptoral structures of these small monogeneans.

### Phylogeny and evolution

Without DNA sequence data of diplectanotrems, it was assumed that the evolutionary specialization towards an endoparasitic lifestyle must have happened multiple times separately, for freshwater and marine taxa [5]. With adaptation to the oesophageal folds instead of the gill filaments for attachment, host specificity seems to reduce. This might either be explained by the probably common morphology of oesophageal epithel cells within various fish species compared to the specialized and differentiated gill rakers and especially filaments. Another factor for decreasing host specificity might be the environmental change from living outside the fish (marine; changing abiotical factors such as temperature, salinity, current, oxygen, light) to inside the fish with a constant physiology. We suggest that adaptation from a highly differentiated site (gill filaments) towards a more general site (stomach) can go along with decreasing host specificity. In the case of diplectanotrem monogeneans, the site change has three benefits. Firstly, the newly explored oesophageal folds are neither utilized by other parasitic worms nor by fish parasitic crustaceans, thus they represent a niche only for diplectanotrems, and resource competition is minimized. Secondly, with exploration of the new habitat, many more fish species and families become potential hosts with empty niches, thus spreading of the diplectanotrems can be maximized. Thirdly, because the host species of *Pseudempleurosoma* spp. appear coastal, often reef-associated, and close to cleaning stations where cleaner fish or shrimp feed on ectoparasites, the new habitat minimizes possible predation.

According to our phylogenetic analysis, *P*. *haywardi* sp. nov. is a close relative of endoparasitic freshwater monogeneans of *Enterogyrus*, distributed in continental Africa and South East Asia. Cichlid fishes originate from Madagascar, and *Enterogyrus* has been associated with cichlid hosts before the colonization of these two continents [14]. If these genera go back to the same origin, diplectanotrem monogeneans might have developed either already from the very basal freshwater cichlids within historical geographically isolated Madagascar, or from the already advanced African cichlids parasitizing monogeneans, subsequently spreading from Africa into the Indo-Pacific region, with the freshwater forms *Enterogyrus* and the marine diplectanotrems. Most interestingly, while the freshwater endoparasitic monogeneans have been reported to be specific to their cichlid hosts, development into the marine forms reduced the host specificity and allowed subsequent worldwide distribution (see Fig 4).

## Conclusion

A new species of endoparasitic monogenean on marine fishes (*Johnius amblycephalus*, *Nibea soldado*, *Otolithes ruber*) from Indonesian waters is described as *Pseudempleurosoma haywardi* sp. nov., based on various unique morphological characteristics as well as on the zoogeographic distribution and hosts. We document different infection rates of the investigated sciaenids, with a prevalence of 46% to 63% (and both *J*. *amblycephalus* investigated were infected), and up the seven worms in the oesophagus of a single fish. We demonstrate that like the other congeners, *P*. *haywardi* sp. nov. is less host specific, and might be found in further of the 39 marine sciaenid species occurring in Indonesia (for list of species, see [26]), or possibly in other fish families, for example sillaginids (compare [22]).

It is noteworthy that the so far recorded host fish species (Tables 2 and 3) are of economic interest, most species have fisheries and/or aquaculture importance, and *Trichiurus lepturus* is of high commercial interest for fisheries [26]. Most of the recorded fish species share similarities concerning their ecology (Table 3), either being reef-associated or benthic over muddy bottoms (except the pufferfish which inhabits (brackish) bays); however, all species are schooling or aggregating. Thus it can be concluded that the genus *Pseudempleurosoma* is somewhat associated to aggregations of fish host species over reefs or muddy bottoms (Table 3).

The phylogeny of marine diplectanotrems is decrypted for the first time, securing the position of these endoparasites and supporting an African freshwater origin. Further phylogenetic analysis of other diplectanotrem genera and of *Montchadskyella*, marine endoparasitic Monogenea of armorheads (Histiopterinae) in Southern Australia, will shed more light on their evolution.

## Supporting information

S1 Table Comparative linear measures for *Pseudempleurosoma haywardi* sp. nov. from the type host *Nibea soldado* (Sciaenidae). All measurements in μm, https://figshare.com/s/75cc37ed9297dc11d983.
(XLSX)

S2 Table Comparative linear measures for *Pseudempleurosoma haywardi* sp. nov. from the additional host *Otolithes ruber* (Sciaenidae). All measurements in μm, https://figshare.com/s/75cc37ed9297dc11d983.
(XLSX)

S1 Fig Confocal photos of the haptor of *Pseudempleurosoma haywardi* sp. nov. Confocal microscopy illustrations of the opisthaptor with hooks, anchors and bars (dorsal bar concave anterior) (**A**), with focus on the inner two (of seven) pairs of hooks, partially overlaid by the ventral anchor with both its bars (**B**) and the same in detail (**C**), https://figshare.com/s/75cc37ed9297dc11d983.
(TIF)

S2 Fig Alignment of sequences. https://figshare.com/s/75cc37ed9297dc11d983.
(TIF)

S1 Permission Permission to use Fig 4. https://figshare.com/s/75cc37ed9297dc11d983.
(TIF)

S1 Calculation Calculation of best fitting model for phylogeny studies. https://figshare.com/s/75cc37ed9297dc11d983.
(XLS)
