# Supplementary figures and images for: Pseudempleurosoma haywardi sp. nov. (Monogenea: Ancyrocephalidae (sensu lato) Bychowsky & Nagibina, 1968): An endoparasite of croakers (Teleostei: Sciaenidae) from Indonesia
Source: PLoS One. 2017 Sep 7;12(9):e0184376. doi: 10.1371/journal.pone.0184376 (PMC5589237; doi:10.1371/journal.pone.0184376)

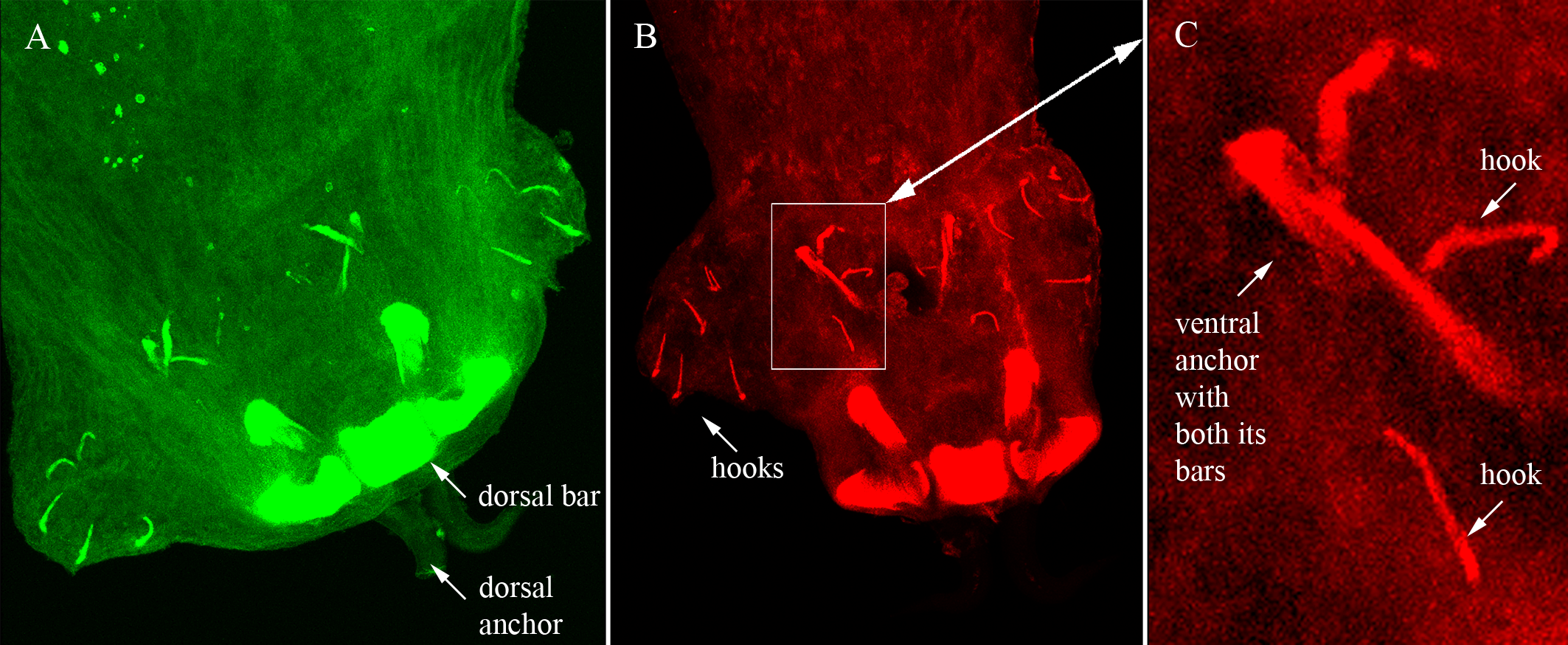

Supplement: S1 Fig — Confocal microscopy illustrations of the opisthaptor with hooks, anchors and bars (dorsal bar concave anterior) (A), with focus on the inner two (of seven) pairs of hooks, partially overlaid by the ventral anchor with both its bars (B) and the same in detail (C), https://figshare.com/s/75cc37ed9297dc11d983. (TIF) [file pone.0184376.s003.tif]

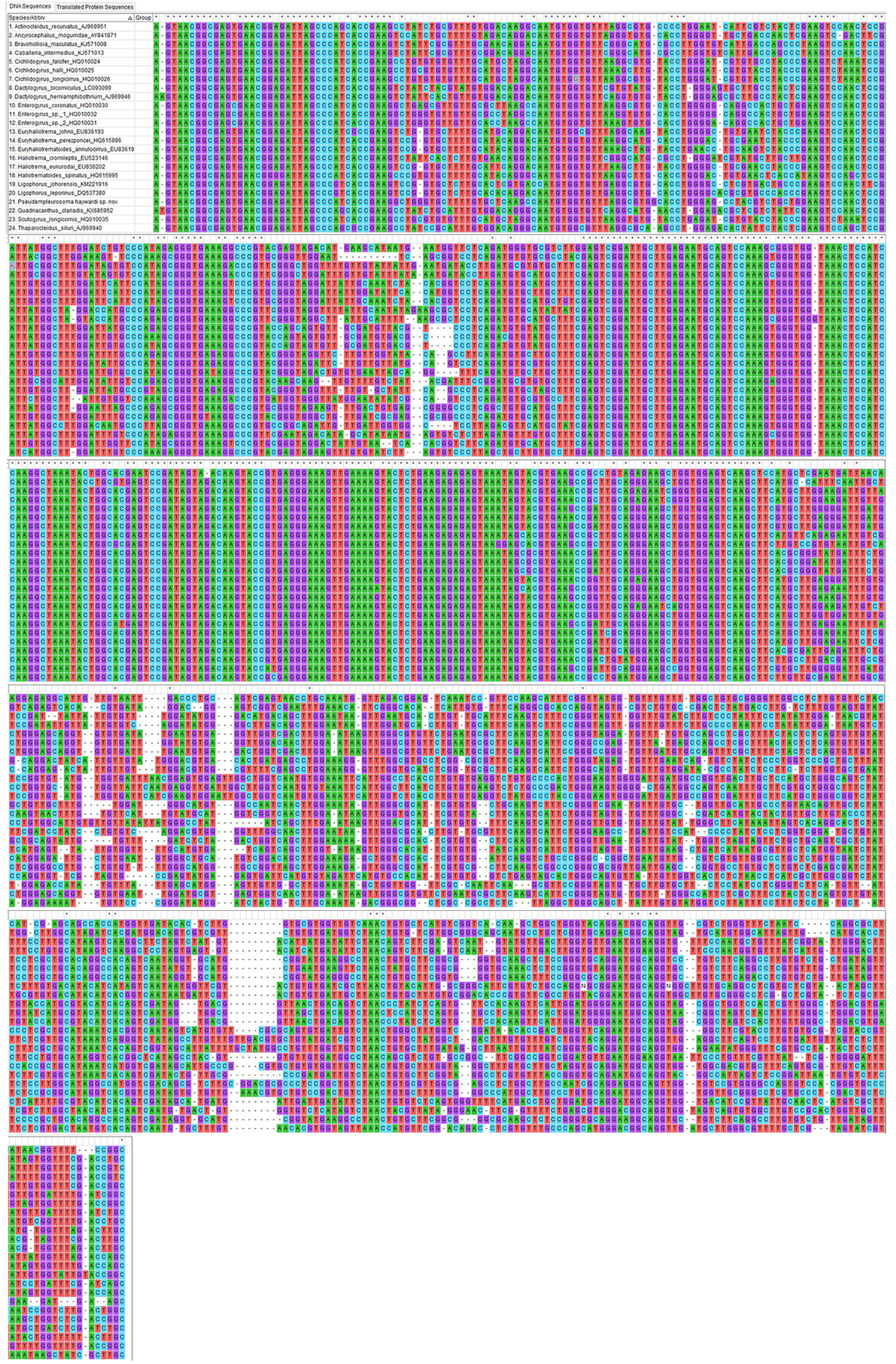

Supplement: S2 Fig — https://figshare.com/s/75cc37ed9297dc11d983. (TIF) [file pone.0184376.s004.tif]

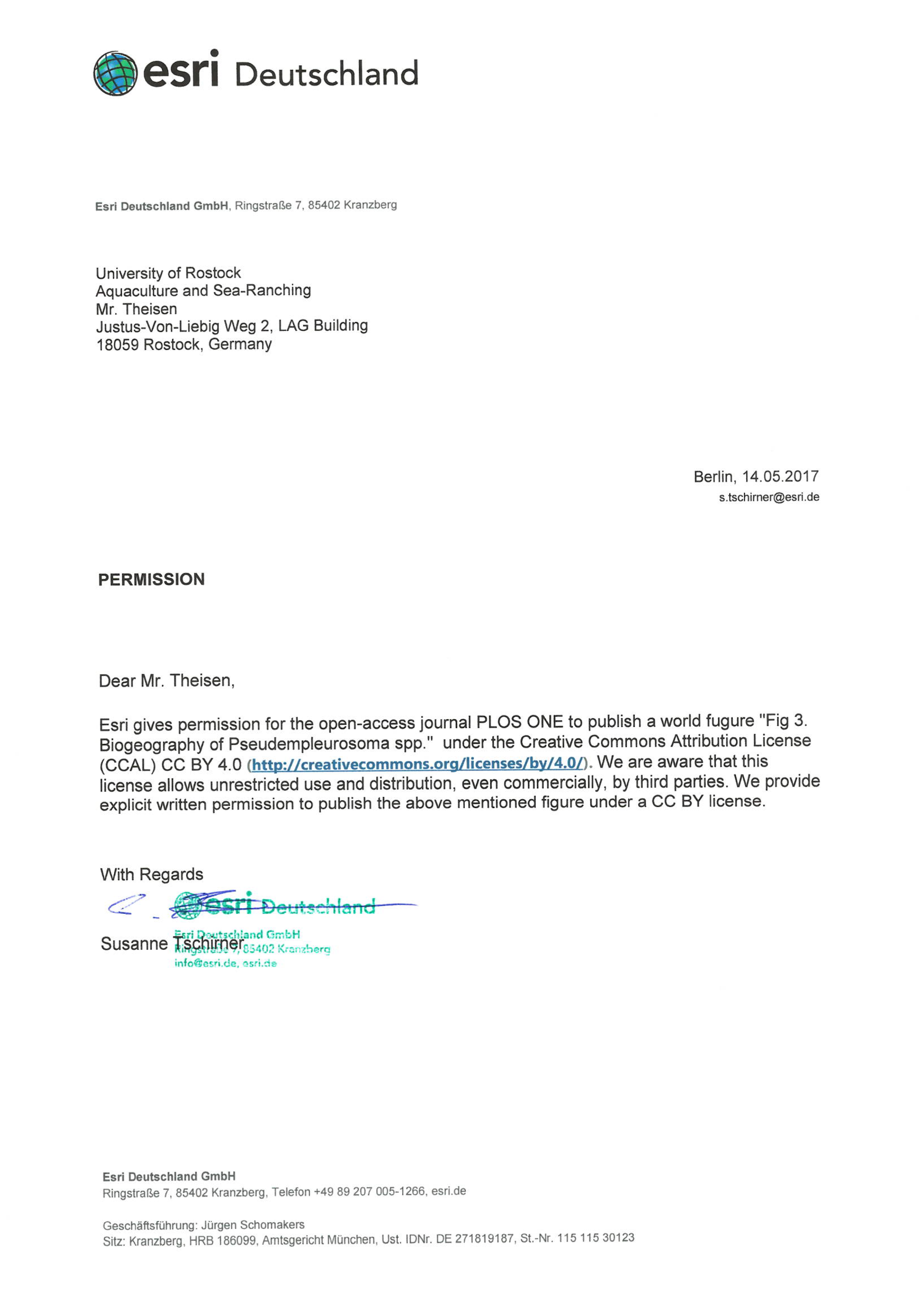

Supplement: S1 Permission — https://figshare.com/s/75cc37ed9297dc11d983. (TIF) [file pone.0184376.s005.tif]
